# Comparison of material properties of heel pad between adults with and without type 2 diabetes history: An *in-vivo* investigation during gait

**DOI:** 10.3389/fendo.2022.894383

**Published:** 2022-08-17

**Authors:** Xiong-gang Yang, Zhao-lin Teng, Zhen-ming Zhang, Kan Wang, Ran Huang, Wen-ming Chen, Chen Wang, Li Chen, Chao Zhang, Jia-zhang Huang, Xu Wang, Xin Ma, Xiang Geng

**Affiliations:** ^1^ Department of Orthopedic Surgery, Huashan Hospital, Fudan University, Shanghai, China; ^2^ Department of Radiology, Huashan Hospital, Fudan University, Shanghai, China; ^3^ Academy for Engineering & Technology, Fudan University, Shanghai, China

**Keywords:** material properties, diabetes, heel pad, dual fluoroscopic system, gait, loading history

## Abstract

**Objective:**

This study was aimed to compare the material properties of heel pad between diabetes patients and healthy adults, and investigate the impact of compressive loading history and length of diabetes course on the material properties of heel pad.

**Methods:**

The dual fluoroscopic imaging system (DFIS) and dynamic foot-ground contact pressure-test plate were used for measuring the material properties, including primary thickness, peak strain, peak stress, stiffness, viscous modulus and energy dissipation ratio (EDR), both at time zero and following continuous loading. Material properties between healthy adults and DM patients were compared both at time zero and following continuous weight bearing. After then, comparison between time-zero material properties and properties following continuous loading was performed to identify the loading history-dependent biomechanical behaviour of heel pad. Subgroup-based sensitivity analysis was then conducted to investigate the diabetes course (<10 years vs. ≥10 years) on the material properties of heel pad.

**Results:**

Ten type II DM subjects (20 legs), aged from 59 to 73 (average: 67.8 ± 4.9), and 10 age-matched healthy adults (20 legs), aged from 59 to 72 (average: 64.4 ± 3.4), were enrolled. Diabetes history was demonstrated to be associated with significantly lower primary thickness (t=3.18, p=0.003**), higher peak strain (t=2.41, p=0.021*), lower stiffness (w=283, p=0.024*) and lower viscous modulus (w=331, p<0.001***) at time zero, and significantly lower primary thickness (t=3.30, p=0.002**), higher peak strain (w=120, p=0.031*) and lower viscous modulus (t=3.42, p=0.002**) following continuous loading. The continuous loading was found to be associated with significantly lower primary thickness (paired-w=204, p<0.001***) and viscous modulus (paired-t=5.45, p<0.001***) in healthy adults, and significantly lower primary thickness (paired-w=206, p<0.001***) and viscous modulus (paired-t=7.47, p<0.001***) in diabetes group. No any significant difference was found when conducting the subgroup analysis based on length of diabetes course (<10 years vs. ≥10 years), but the regression analysis showed that the length of diabetes history was positively associated with the peak strain, at time zero (r=0.506, p<0.050) and following continuous loading (r=0.584, p<0.010).

**Conclusions:**

Diabetes patients were found to be associated with decreased primary thickness and viscous modulus, and increased peak strain, which may contribute to the vulnerability of heel pad to injury and ulceration. Pre-compression history-dependent behaviour is observable in soft tissue of heel pad, with lowered primary thickness and viscous modulus.

## Introduction

Diabetes mellitus (DM) is a 21^st^ century epidemic, affecting about 9.3% of the world’s population at 2019 (1). This figure is estimated to be as high as 10.2% and 10.9% by 2030 and 2045 respectively, according to the 9^th^ International Diabetes Federation (IDF) Conference (1). Diabetic foot ulcer (DFU), as one of the most common, serious and destructive complications, would be experienced by about 25% of the DM patients over their course of disease (2, 3). Despite an advanced team cooperation, many ulcerous foots must be amputated within 4 years due to unsatisfactory treatment effect, comprising up to one-third of the direct expense of diabetic care (4–6). It is estimated that 10% and 19% of the DFU patients would encounter major amputation and minor amputation respectively, within 1 year of diagnosis (7). Although the heel ulcerations are less common than forefoot ulcerations, they are generally more challenging to treat, difficult to heal and associated with larger morbidity and higher costs (8, 9). It is reported that the limb salvage success rate of heel ulcers is 2~3 times less than that of the forefoot ulcers (8). As the first point of contact between the foot and ground during human locomotion, the heel acts as an efficient shock absorber to dampen the impact stress transferred to the skeletal system (10–12). The heel fat pad is histologically composed of honeycombed fat globules formed by clustered fat cells in whorls of fibroelastic septa (13). The intact configurations of the adipocyte cluster and fibrous envelop are necessary for heel pad to resist the external compressive loads during the stance phase of gait cycle (11, 13, 14). However, the histomorphology of the heel pad would be significantly altered, including breaking of collagen bundles and fragmentation of elastin strands in septa, and relative shrinking of adipocytes, in pathological condition of diabetes (10, 11, 15–17). These alterations, subsequently, would lead to further modifications on the material properties, causing increased stiffness of septa, decreased damping ability, and increased vulnerability of tissue to injury (10, 13, 15–18).

To date, researchers have developed plenty of footwear and custom insole designs to prevent the ulceration of the plantar soft tissue in diabetes. These designs, predominately, depended on the premise that the elevated barefoot pressure (or peak stress) is the main cause of diabetic plantar ulceration (19–21). Subsequently, the major goal of the footwear and insole is to reduce or redistribute the pressure on the plantar surface at locations with risk of ulceration. Additionally, the pressure relieving effect has been widely adopted as the primary index to evaluate the treatment efficacy (19–21). However, diabetes patients sometimes develop ulcers at the locations with normal pressure, or the elevated peak pressure is available in some healthy adults (22, 23). Veves et al. (24) reported that only 38% of the DFU locations matched with the peak pressure areas in plantar surface. Healy et al. (25) indicated that over 42% of the diabetic amputations were caused by improper footwear. To accurately identify the comprehensive mechanical parameters more than peak pressure, therefore, is essential for guiding the designing of footwear and insole to compensate the atrophy and degeneration of diabetic plantar tissue. Several methods have been established to investigate the viscoelastic material properties of heel pad through either *in vitro* cadaver or *in vivo* volunteer experiments (14, 26–37). The uniaxial compression and stress-relaxation experiments with *in-vitro* tissue cut from cadaveric plantar pad was firstly performed to test the material properties (14, 27, 28). These tests, nevertheless, have been proven to overestimate the stiffness (six times higher) and underestimate the energy dissipation ratio (EDR, three times lower) compared to *in-vivo* testing, because of the inclusion of entire lower leg for *in vivo* tests (29–31). More recently, some novel *in vivo* methods, such as indentation test (26, 32), drop impact test (29), ballistic pendulum (33) and ultrasound elastography approach (34, 35), have been widely applied to quantify the static or quasi-static material properties of plantar soft tissue. These methods, nevertheless, are not able to replicate the actual loading conditions experienced by the heel when contacting with the ground during dynamic gait cycle, as the heel pad is loaded at a fixed position without movement. To overcome this limitation, De Clercq et al. (36) and Gefen et al. (37) developed an innovative method by incorporating of the fluoroscopy (cine-radiography) and simultaneous pressure test plate beneath the foot to measure the *in vivo* material properties during dynamic gait (36, 37). Up until then, it was possible to dynamically investigate the material properties of heel pad. However, this method was based on two-dimensional fluoroscopy, which would lead to bias on measured strain due to varying shooting angle. Instead, the dual fluoroscopic imaging system (DFIS) could capture two perpendicularly intersected images for reconstructing a three-dimensional structure, and has been recently applied in many situations to help investigate the structural and locational indexes. In a previous preliminary study, we have established a novel system for testing the material properties of heel pad, and performed pilot investigation within several healthy adults (38).

In this study, we set out to: (i) identify the material properties of heel pad in DM patients; (ii) compare the material properties of heel pad between DM patients and healthy adults; (iii) perform subgroup analysis according to diabetes history with the aim of comparing the material properties between DM patients with <10 and ≥10 years of DM history; (iv) investigate the effect of continuous compression load on the material properties of heel pad.

## Materials and methods

### Inclusion of subjects

In accordance with the Declaration of Helsinki, and upon attaining the ethical approval from the Institutional Review Board of Huashan Hospital, Fudan University, 10 type II DM subjects and 10 age-matched healthy adult subjects were enrolled. The DM subjects fulfill the Guidelines for the Prevention and Control of Type 2 Diabetes in China (2017 edition) (39). All subjects received their informed consent at the time before examination. All subjects had palpable pulse at their dorsalis pedis and posterior tibial arteries. Participants with pathological conditions other than diabetes that could affect the properties of heel pad, including heel pain, rheumatoid arthritis, foot deformity, dysvascula of foot, history of foot surgery, and trauma of foot, were excluded. Each subject received CT scan on foot before the experiment, for building models of calcaneus that were used in 2D-3D registration with Mimics Medical 21.0 (Materialize, Belgium).

### Instrumentation and experiment design

**Figure 1:**
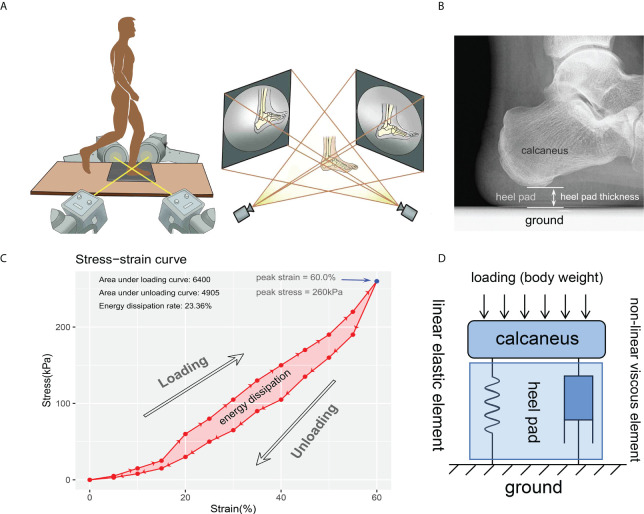
Diagrams for the experiment instrument and data process. **(A)** instrumentation including two orthogonally placed fluoroscopes and dynamic foot-ground contact force plate embedded in the custom gait platform. Using this instrumentation, two 2-D images at each time were obtained for reconstructing the 3-D model and investigate the heel pad thickness. **(B)** thickness of the heel pad was defined as the perpendicular distance between the ground and the base of the calcaneus. **(C)** a representative stress-strain curve depicting a cycle of loading and unloading. The most-right blue point indicates the peak stress (260kPa) and peak strain (60.0%). The energy dissipation was defined as the area between the loading and unloading curves (the light red area), and the energy dissipation rate (23.36%) was defined as the ratio between energy dissipation and area under loading curve. **(D)** modified Kelvin-Voigtviscoelastic model composed of parallelly connected linear elastic element and non-linear viscous element representing the viscoelastic characteristic of heel pad.

A diagram of the equipment is presented in **Figure 1A**. Two C-arm fluoroscopes (BV Pulsera, Phillips Medical, USA) placed orthogonally were utilized for capturing the *in vivo* compressive strain of the heel pad, with pre-set frame rate of 50 Hz, resolution of 1024×1024 pixels and beam energy setting of 75kV•40mA. At beginning of the experiment, a single cubic and a pair of retiform custom-made calibrators were used for obtaining calibration images, which were then used for calibrating the distortions of fluoroscopies before model-image registration. At the same time, a dynamic foot-ground contact pressure-test plate (zebris PDM-XS, 570*400*15mm, Germany) was embedded in the custom gait platform (with length of 3.5m and width of 0.8m) to continuously record the evolution of compressive pressure.

A cube calibrator and a pair of retiform calibrators matching the biplane were placed at the intersection of the two fluorescent projections, and placed on the two receivers of the fluoroscopes, respectively. Following gathering of the calibration images with the aid of software XMAlab (https://bitbucket.org/xromm/xmalab/), subjects were trained to walk barefooted on the gait platform with the aid and protection of researchers, until they could locomote deftly and stably without any aid. To eliminate the impact of strain rate on the material properties of heel fat pad, the subjects were required to move with an approximate gait velocity of 1.0 m/s by visual inspection. A “two-step” gait cycle was performed for all subjects, with the second step striking at a marked position on the force plate with the heel to maintain the location of the tested foot to be in the view of fluoroscopes. Both the left and right heels were measured using the identical procedures. After all subjects were familiar with the experiment procedure, their foots were kept in relaxation and free of load for one hour. Then, the subjects were taken back to test the time-zero (i.e., the status without continuous loading on the heel pad) material properties. Next, with the aim of investigating the impact of loading history on material properties, the subjects were required to keep their foots on continuous loading by sustaining standing or roaming in the laboratory room for 15 minutes, after which the material properties of the heel pad were measured.

### Data processing

The data obtained by DFIS were handled with software Rhinoceros 5.0 (Robert McNeel & Associates, Washington, USA), to get the time-dependent strain rate of heel pad. The time-matched data recorded by contact force plate were exported as xml file to calculate the heel-ground contact stress. As shown in **Figure 1B**, the thickness of the heel pad was measured on each continuous frame, to figure out the perpendicular distance between the ground and the base of the calcaneus. The “primary thickness” was defined as the thickness measured on the frame where skin of heel initially contacts with force plate. Strain (Ɛ_c_) was change in thickness divided by primary thickness. Stress (б_c_) was defned as heel-ground contact force of heel area divided by acreage in each frame. Stress-strain curve, depicting a cycle of loading and unloading process of heel pad, was then generated using strain values and stress values matched according to time points. A case of stress-strain curve is shown in **Figure 1C**. The “peak stress” and “peak strain” were defined as the stress and strain at the most right point in stress-strain curve. The stress-strain data of each foot were fitted to the Kelvin-Voigt model (б_c_=−E_c_ - ηƐ_c_έ_c_), and the Young’s modulus (E) and viscous modulus (η) were obtained according to the least squares method (**Figure 1D**). The energy dissipation was defined as the area in the hysteresis loop bounded by the loading and unloading curves, and the EDR was expressed as the ratio of energy dissipation and the area under the loading curve.

### Statistical analyses

Continuous variables were presented as mean ± SD (standard deviation) in case of meeting the normality distribution, or presented as median and range in case of not meeting normality distribution. Age and BMI between healthy and diabetes groups were compared with student-t test as fulfilling the normality and homoscedasticity.

When comparing the material properties (time zero, following continuous loading, and difference between two statuses) between healthy adults and DM patients, student-t test (meet the normal distribution and homoscedasticity assumptions), Welch-t test (meet the normal distribution and but not homoscedasticity) or Wilcoxon rank sum test (not meet the normal distribution assumption) were selected for statistical analyses. When comparing the material properties (healthy adults and DM patients) between two loading statuses, paired-t test (difference between two statuses must meet normal distribution) or paired-Wilcoxon test (difference between two statuses not meet normal distribution) were selected for statistical analyses.

Subgroup analysis was performed according to the diabetes history, including the groups with diabetes course of <10 years and ≥10 years. One-way analysis of variance (ANOVA, meet the normal distribution and homoscedasticity assumptions), Brown-Forsythe test (meet the normal distribution and but not homoscedasticity), or Kruskal-Wallis test (H test, not meet the normal distribution assumption) were performed to compare the difference among healthy group and two diabetes groups. After that, *post hoc* analyses, including SNK-q (for ANOVA), Tamhane’s T2 (for Brown-Forsythe test) and Dunn’s (for H test) tests were selected to conduct pair-wise comparison for the three groups. For the two subgroups of diabetes subjects and the subgroups of left and right legs, the material properties at time-zero and following continuous loading were compared with paired-t test (difference between two statuses meet normal distribution) or paired-Wilcoxon test (difference between two statuses not meet normal distribution) were selected for statistical analyses.

In healthy adults, correlation matrices were generated for properties measured at time zero and following continuous loading, and for the differences of properties measured at two statuses. In diabetes subjects, correlation matrices were generated for properties measured at time zero and following continuous loading, and for the differences of properties measured at two statuses, with subgroup analysis for the length of diabetes course (either <10 or ≥10 years). Pearson’s correlation analysis was used to detect the correlation between continuous variables in matrices, and correlation efficient (R) was calculated to depict the magnitude of the correlativity.

The above statistical analyses were conducted with R version 4.0.5 (Foundation for Statistical Computing, Vienna, Austria). Significance level was defined as p value of less than 0.050. The normality test and homogeneity test of variances were conducted with Shapiro-Wilk test (W test, p<0.05 indicates significant non-normality) and modified Bartlett’s test (p<0.05 indicates significant non-homoscedasticity).

## Results

### Baseline characteristics

The baseline characteristics of the enrolled patients are available in **Table 1**. A total of 10 type II DM subjects (9 male and 1 female, 20 legs), aged from 59 to 73 (average: 67.8 ± 4.9), and 10 age-matched healthy adult subjects (9 male and 1 female, 20 legs), aged from 59 to 72 (average: 64.4 ± 3.4), were enrolled. The median value of the length of diabetes course was 9.5 (range: 2~25) years in diabetes group, with five subjects have a diabetes history of <10 years (mean: 4.8 ± 2.8 years) and five ≥10 years (mean: 16.2 ± 5.9 years). No significant difference was presented for age (t=1.80, p=0.088) and BMI (t=0.43, p=0.676) between healthy group and diabetes group. Between the subgroups with diabetes courses of <10 and ≥10 years, similar age (t=0.122, p=0.906) and BMI (t=0.11, p=0.917) were demonstrated.

**Table 1:** Baseline characteristics of the enrolled patients.

Variables	Normal group (n=10)	Diabetic foot group			p value (normal vs. whole diabetic foot)	p value (diabetes history <10y vs. diabetes history ≥10y)
		Whole group (n=10)	Diabetes history (<10 y, n=5)	Diabetes history (≥10 y, n=5)		
**Age**	64.4±3.4	67.8±4.9	67.6±3.7	72 (range: 59~73)^#^	T=1.80, p=0.088	T=0.122, p=0.906
**BMI**	24.1±2.2	24.7±3.9	25.4±3.5	25.1±4.3	T=0.43, p=0.676	T=0.11, p=0.917
**Length of diabetes history**	NA	9.5 (range: 2~25)^#^	4.8±2.8	16.2±5.9	NA	NA
**Sex**						
Male-n(%)	9	9	4	5	NA	NA
Female-n(%)	1	1	1	0

^#^data are presented with the median values as well as the minimum-to-maximum ranges, as they don’t follow a normal distribution. NA, not applicable; BMI, body mass index.

### Impact of diabetes and loading history on material properties of heel pad

**Figure 2:**
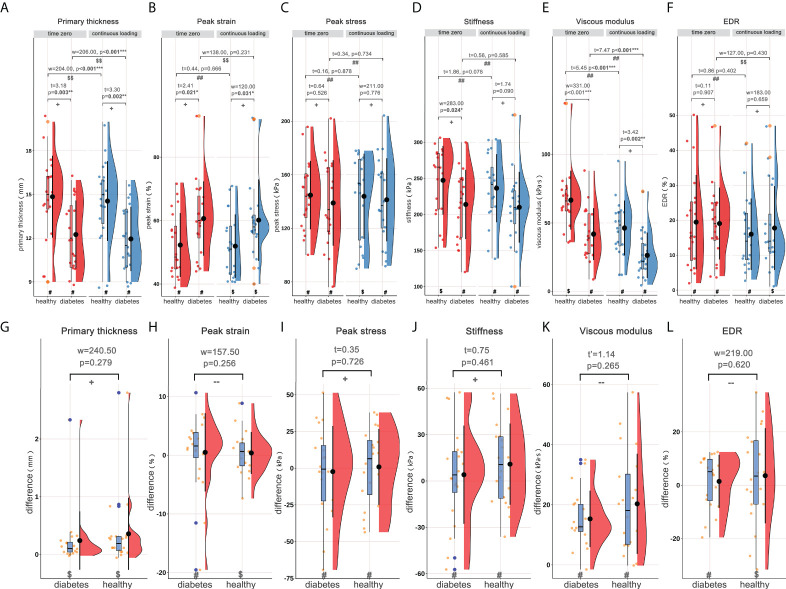
Rain-cloud plot depicting the material properties of heel pad, including primary thickness, peak strain, peak stress, stiffness, viscous modulus and EDR. **(A–F)** the material properties of heel pad in healthy and diabetes groups at time zero and following continuous loading. **(G–L)** the differences of material properties of heel pad between two loading statuses in healthy and diabetes groups. ^#^, data follow the normality distribution; ^$^, data didn’t follow the normality distribution; ^##^, the difference between two loading statuses follow the normal distribution; ^$$^, the difference between two loading statuses didn’t follow the normal distribution; +, meet the homoscedasticity assumption; -, didn’t meet the homoscedasticity assumption. “t”, “t’”, and “w” represent the statistical effect sizes for T test, Welch’s T test, and Wilcoxon test, respectively. Error bar represents the mean value and standard deviation; box plot represent the median value and quartiles. EDR= energy dissipation rate. *, p<0.050; **p<0.010; ***p<0.001.

Using the DFIS incorporated with force plate, time-dependent strain and stress applied to heel pad were concurrently obtained. The summaries of the material properties of heel pad, including the mean/median values of properties at time zero and following continuous loading and the differences between two loading statuses, are presented in **Table 2**. **Figure 2** shows the rain-cloud plot depicting the material properties of heel pad in healthy and diabetes subjects. Diabetes history was demonstrated to be associated with significantly lower primary thickness (**Figure 2A**, t=3.18, p=0.003**), higher peak strain (**Figure 2B**, t=2.41, p=0.021*), lower stiffness (**Figure 2D**, w=283, p=0.024*) and lower viscous modulus (**Figure 2E**, w=331, p<0.001***) at time zero, and significantly lower primary thickness (**Figure 2A**, t=3.30, p=0.002**), higher peak strain (**Figure 2B**, w=120, p=0.031*) and lower viscous modulus (**Figure 2E**, t=3.42, p=0.002**) following continuous loading. The continuous loading history was found to be associated with significantly lower primary thickness (**Figure 2A**, paired-w=204, p<0.001***) and viscous modulus (**Figure 2E**, paired-t=5.45, p<0.001***) in healthy adults, and significantly lower primary thickness (**Figure 2A**, paired-w=206, p<0.001***) and viscous modulus (**Figure 2E**, paired-t=7.47, p<0.001***) in diabetes group. There was no any significant difference when comparing the difference of material properties between two groups of subjects.

**Table 2:** Summaries of the material properties of plantar soft tissue at heels of normal adults and adults with diabetic foot.

Properties	Normal group (n=20)	Diabetic foot group		
		Whole group (n=20)	Diabetes history (<10 y, n=10)	Diabetes history (≥10 y, n=10)
**Time zero**				
-Primary thickness (mm)	14.85±2.81	12.25±2.34	12.69±2.16	12.54±2.62
-Peak strain (%)	52.30±10.09	60.65±11.73	58.80±10.44	62.50±13.18
-Peak stress (kPa)	144.80±25.56	138.95±31.93	135.70±35.40	142.20±29.59
-Young’s modulus (kPa)	265.50 (range: 154.79~306.28)^#^	214.39±48.04	206.50±43.08	221.30±53.69
-Viscous modulus (kPa۰s)	66.59 (range: 36.70~137.97)^#^	42.28±18.93	36.20±14.37	47.60±21.74
-EDR (%)	19.45±13.49	19.05±10.29	18.00±8.35	20.10±12.30
**Following continuous loading**				
-Primary thickness (mm)	14.55±2.74	11.95±2.21	11.90±2.13	12.00±2.40
-Peak strain (%)	51.55 (range: 41.09~71.85)^#^	58.61 (range: 40.64~92.92)^#^	57.23±8.72	58.96 (range: 48.12~92.92)^#^
-Peak stress (kPa)	153.74 (range: 90.80~178.55)^#^	141.82±30.95	138.10±32.63	144.60±30.71
-Young’s modulus (kPa)	236.40±47.21	209.95±49.04	204.60±34.58	215.30±61.79
-Viscous modulus (kPa۰s)	46.30±19.99	26.45±16.55	23.10±11.12	29.80±20.72
-EDR (%)	16.00±10.23	14.44 (range: 1.20~47.52)^#^	14.20±8.28	21.30±14.41
**Difference between time-zero and continuously loaded heel pads**				
-Primary thickness (mm)	0.19 (range: -0.06~2.80)^#^	0.13 (range: -0.02~2.33)^#^	0.17 (range: 0.04~2.33)^#^	0.09±0.11
-Peak strain (%)	0.39±3.57	1.57 (range: -19.53~10.65)^#^	2.36±5.79	1.11 (range: -19.53~6.99)^#^
-Peak stress (kPa)	0.80±25.41	-2.35±30.72	-2.22±38.40	-2.48±23.86
-Young’s modulus (kPa)	10.75±25.97	3.95±31.48	1.86±33.37	6.25±31.84
-Viscous modulus (kPa۰s)	19.85±16.72	15.00±9.23	12.92±9.89	17.72±8.71
-EDR (%)	3.59±17.83	5.10 (range: -19.56~12.48)^#^	3.98±8.74	-1.10±10.88

^#^data are presented with the median values as well as the minimum-to-maximum ranges, as they don’t follow a normal distribution.

### Subgroup analyses basing on course of diabetes and side of leg

**Figure 3:**
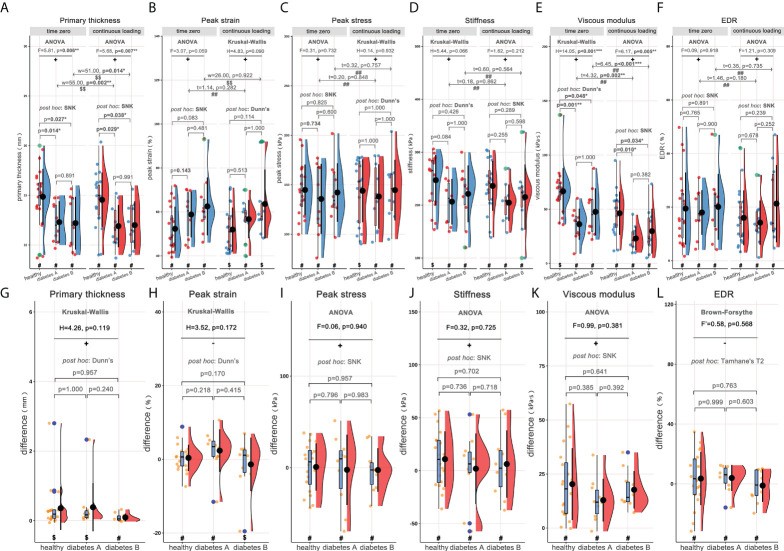
Rain-cloud plot for subgroup analysis based on length of diabetes course (diabetes A: <10years; diabetes B: ≥10years), depicting the material properties of heel pad, including primary thickness, peak strain, peak stress, stiffness, viscous modulus and EDR. **(A–F)** the material properties of heel pad in three subgroups (healthy, diabetes A and diabetes B) at time zero and following continuous loading. **(G–L)** the differences of material properties of heel pad between two loading statuses in three subgroups. ^#^, data follow the normality distribution; ^$^, data didn’t follow the normality distribution; ^##^, the difference between two loading statuses follow the normal distribution; ^$$^, the difference between two loading statuses didn’t follow the normal distribution; +, meet the homoscedasticity assumption; -, didn’t meet the homoscedasticity assumption. “F”, “F’”, “H”, “t” and “w” represent the statistical effect sizes for ANOVA, Brown-Forsythe test, Kruskal-Wallis test, T test, and Wilcoxon test, respectively. Error bar represents the mean value and standard deviation; box plot represent the median value and quartiles. ANOVA= analysis of variance; EDR= energy dissipation rate. *, p<0.050; **p<0.010; ***p<0.001.

Subgroup analyses were performed basing on length of diabetes course (<10 and ≥10 years, marked as diabetes A and diabetes B groups respectively), and the rain-cloud plot is available in **Figure 3**. Generally, significantly different primary thickness (**Figure 3A**, ANOVA: F=5.81, p=0.006**; ANOVA: F=5.68, p=0.007**), and viscous modulus (**Figure 3E**, Kruskal-Wallis: H=14.05, p<0.001***; ANOVA: F=6.17, p=0.005**) were demonstrated both at time zero and following continuous loading. *Post hoc* analyses demonstrated significantly higher primary thickness (**Figure 3A**, time zero: SNK, p=0.014*/p=0.027*; following continuous loading: SNK, p=0.029*/p=0.038*) and viscous modulus (**Figure 3E**, time zero: Dunn’s, p=0.001**/p=0.048*; following continuous loading: SNK, p=0.010*/p=0.034*) both at time zero and following continuous loading, for healthy subjects when compared with diabetes A and diabetes B groups. When comparing the material properties between two loading statuses, the continuous loading was found to be associated with significantly lower primary thickness (**Figure 3A**, healthy adults: paired-w=55, p=0.002**; diabetes subjects: paired-w=51, p=0.014*) and viscous modulus (**Figure 3E**, healthy adults: paired-t=4.32, p=0.002**; diabetes subjects: paired-t=6.45, p<0.001***) both for healthy adults and diabetes subjects. No any significant difference was found when comparing the difference of material properties among three subgroups of subjects.

The rain-cloud plot depicting the subgroup analysis comparing the material properties of left and right sides is available in **Supplementary Figure 1**. The material properties at time zero (A-F) and following continuous loading (G-L) were compared, and no any significant difference was detected between two groups.

### Correlation analysis for material properties of heel pad

**Figure 4:**
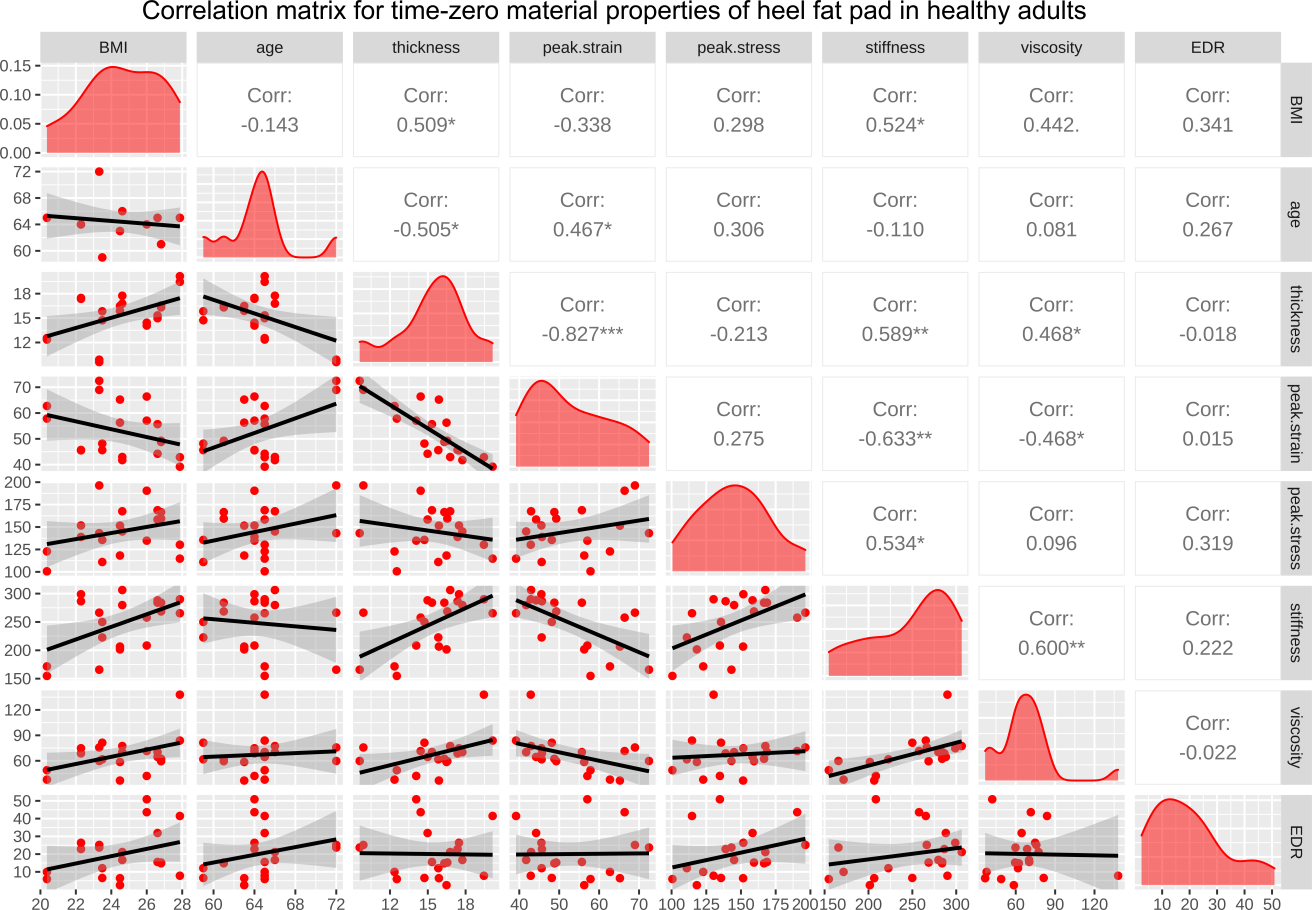
The correlation matrix for time-zero BMI, age, primary thickness, peak strain, peak stress, stiffness, viscous modulus, and EDR of heel in healthy adults. The values displayed in the right-upper triangle represent the Pearson’s correlation coefficients (R values). The lower-left triangle displays the scatter plots and regression lines. The plots on the diagonal line present the distribution density of the variables in the matrix. BMI: body mass index; EDR= energy dissipation rate. P values:.p<0.100, *p<0.050, **p<0.010, ***p<0.001.

**Figure 5:**
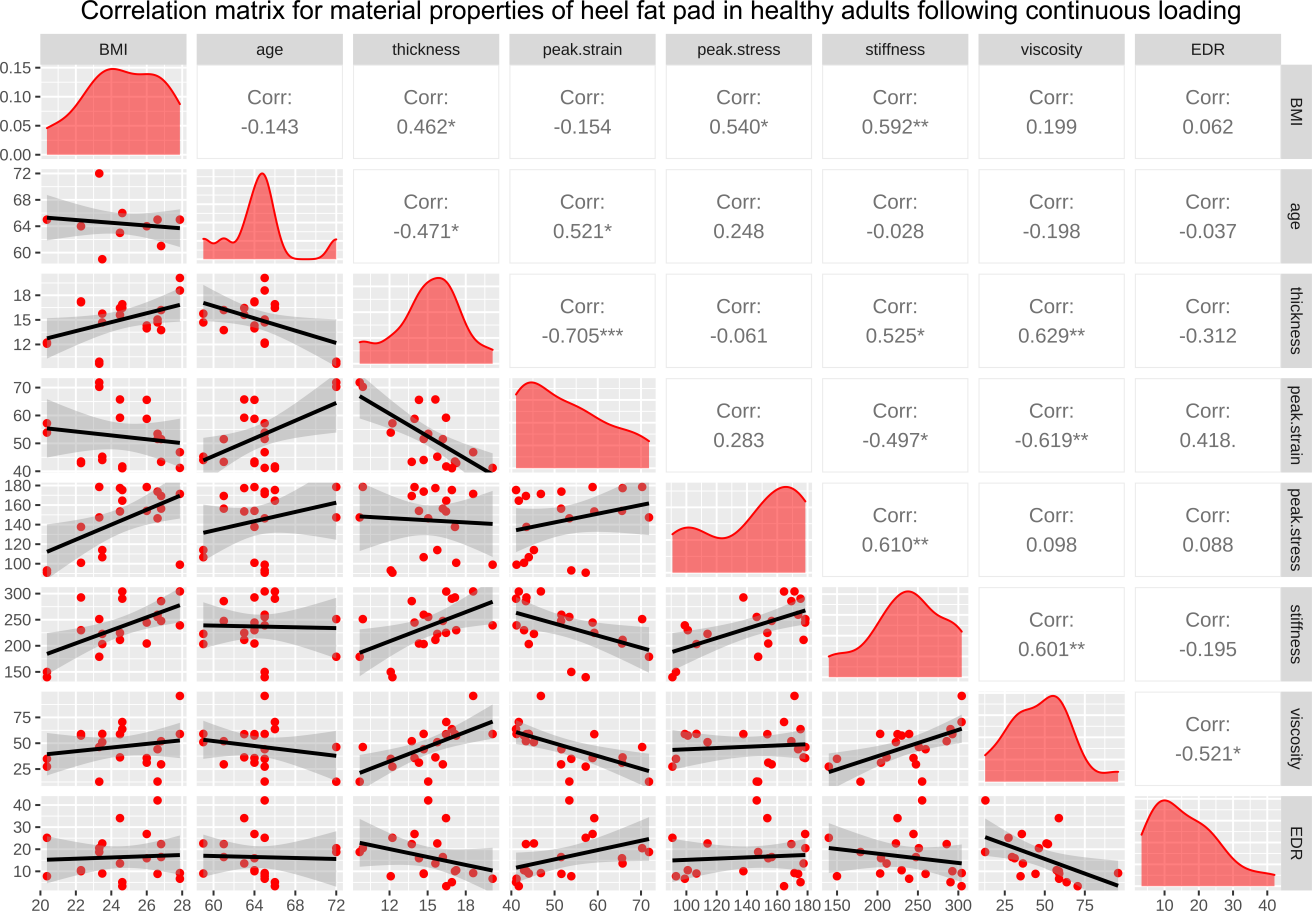
The correlation matrix for BMI, age, primary thickness, peak strain, peak stress, stiffness, viscous modulus, and EDR of heel in healthy adults following continuous loading. The values displayed in the right-upper triangle represent the Pearson’s correlation coefficients (R values). The lower-left triangle displays the scatter plots and regression lines. The plots on the diagonal line present the distribution density of the variables in the matrix. BMI: body mass index; EDR= energy dissipation rate. P values:.p<0.100, *p<0.050, **p<0.010, ***p<0.001.

The correlation matrices for BMI, age, and the properties of heel in healthy adults at time zero and following continuous loading are available in **Figures 4**, **5**, respectively. As a result, at time zero, primary thickness was shown to be significantly correlated with BMI (R=0.509, p<0.050*) and age (R=-0.505, p<0.050*); peak strain was significantly correlated with age (R=0.467, p<0.050*) and primary thickness (R=-0.827, p<0.001***); stiffness was significantly correlated with BMI (R=0.524, p<0.050*), primary thickness (R=0.589, p<0.010**), peak strain (R=0.-0.633, p<0.010**) and peak stress (R=0.534, p<0.050*); viscous modulus was significantly correlated with primary thickness (R=0.468, p<0.050*), peak strain (R=-0.468, p<0.050*) and stiffness (R=0.600, p<0.010**). Following continuous loading, primary thickness was shown to be significantly correlated with BMI (R=0.462, p<0.050*) and age (R=-0.471, p<0.050*); peak strain was significantly correlated with age (R=0.521, p<0.050*) and primary thickness (R=-0.705, p<0.001***); peak stress was significantly correlated with BMI (R=0.540, p<0.050*); stiffness was significantly correlated with BMI (R=0.592, p<0.010**), primary thickness (R=0.525, p<0.050*), peak strain (R=0.-0.497, p<0.050*) and peak stress (R=0.610, p<0.010*); viscous modulus was significantly correlated with primary thickness (R=0.629, p<0.010**), peak strain (R=-0.619, p<0.010**) and stiffness (R=0.601, p<0.010**); EDR was significantly correlated with viscous modulus (R=-0.521, p<0.050*).

**Figure 6:**
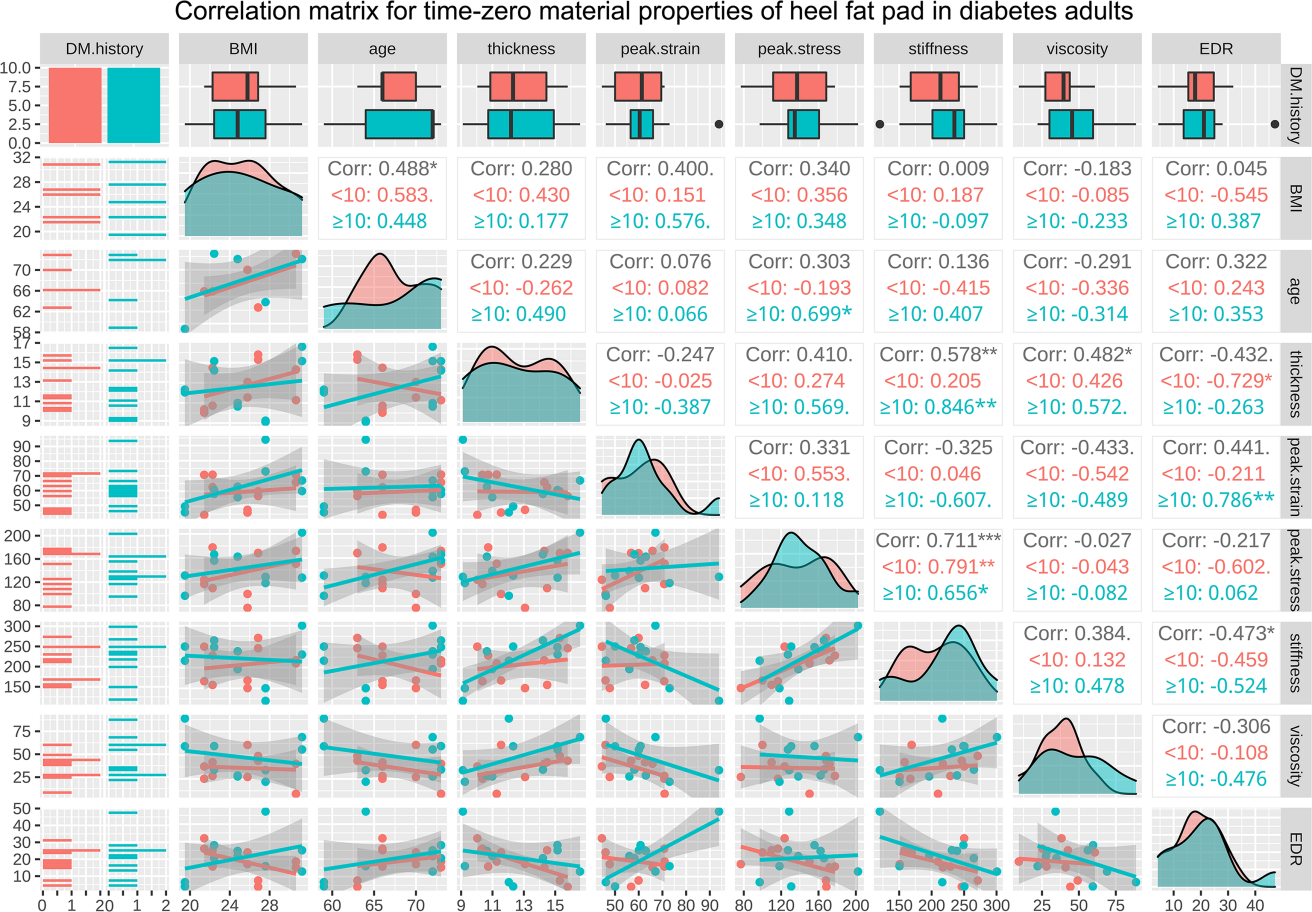
The correlation matrix for time-zero BMI, age, primary thickness, peak strain, peak stress, stiffness, viscous modulus, and EDR of heel in diabetes subjects. The values displayed in the right-upper triangle represent the Pearson’s correlation coefficients (R values). The lower-left triangle displays the scatter plots and regression lines. The plots on the diagonal line present the distribution density of the variables in the matrix. Subgroup analysis was conducted basing on the diabetes course (<10 years and ≥10 years), and red colour represents subgroup of <10 years while cyan colour represents subgroup of ≥10 years. BMI: body mass index; EDR= energy dissipation rate. P values:.p<0.100, *p<0.050, **p<0.010, ***p<0.001.

**Figure 7:**
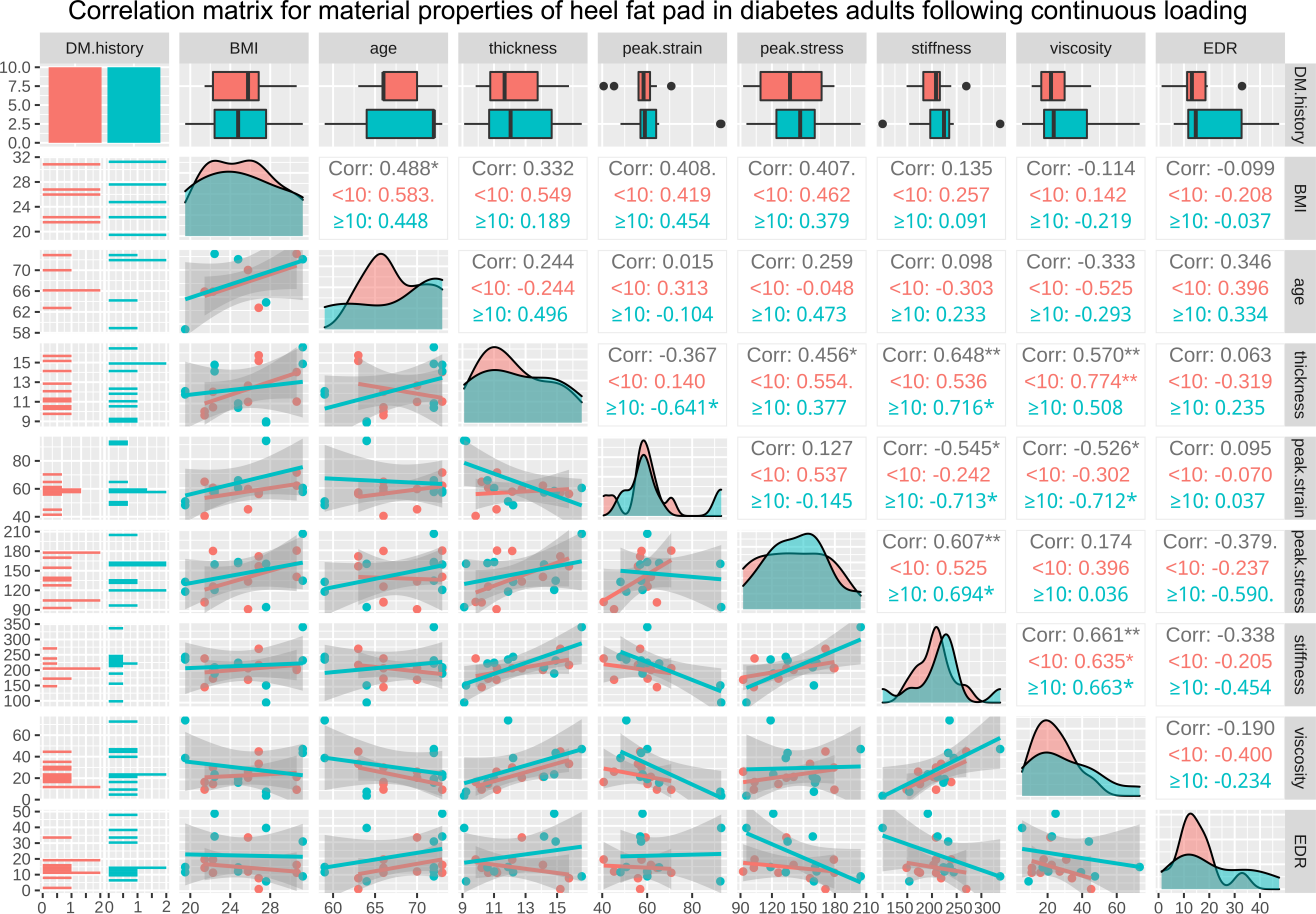
The correlation matrix for BMI, age, primary thickness, peak strain, peak stress, stiffness, viscous modulus, and EDR of heel in diabetes subjects following continuous loading. The values displayed in the right-upper triangle represent the Pearson’s correlation coefficients (R values). The lower-left triangle displays the scatter plots and regression lines. The plots on the diagonal line present the distribution density of the variables in the matrix. Subgroup analysis was conducted basing on the diabetes course (<10 years and ≥10 years), and red colour represents subgroup of <10 years while cyan colour represents subgroup of ≥10 years. BMI: body mass index; EDR= energy dissipation rate. P values:.p<0.100, *p<0.050, **p<0.010.

The correlation matrices for length of diabetes course, BMI, age, and the properties of heel in diabetes subjects at time zero and following continuous loading are available in supplementary **Figures 2**, **3**, respectively. The regression analysis results showed that the length of diabetes history was positively associated with the peak strain, at time zero (r=0.506, p<0.050) and following continuous loading (r=0.584, p<0.010). The correlation matrices for BMI, age, and the properties of heel in diabetes subjects at time zero and following continuous loading, with subgroup analyses based on course of diabetes, are available in **Figures 6**, **7**, respectively. At time zero, stiffness was significantly correlated with primary thickness (R=0.578, p<0.010**), peak stress (R=0.711, p<0.001***) and EDR (R=-0.473, p<0.050*); primary thickness was significantly correlated with viscous modulus (R=0.482, p<0.050*). Following continuous loading, stiffness was significantly correlated with primary thickness (R=0.648, p<0.010**), peak strain (R=-0.545, p<0.050*) and peak stress (R=0.607, p<0.010**); viscous modulus was significantly correlated with primary thickness (R=0.570, p<0.010**), peak strain (R=-0.526, p<0.050*) and stiffness (R=0.661, p<0.010**).

## Discussion

### Main findings

In this study, using our previously reported novel method, further investigation of the material properties of heel pad in diabetes patients was conducted, with combination of the DFIS and force plate. The main findings of the current study include: (i) diabetes disease was associated with decreased primary thickness and viscous modulus, and increased peak strain both at time zero and following continuous loading; (ii) loading history (continuous weight-bearing) was related to decreased primary thickness and viscous modulus both for healthy adults and diabetes subjects; (iii) length of diabetes course has no obvious impact on the material properties of heel pad.

### Material properties of heel pad in DM patients

The biomechanical changes in DM patients have been widely investigated with various methods (9, 28, 35, 40–46). However, it remains a difficulty to accurately measure the material properties of heel pad during normal gait with non-invasive approach. To replicate the stance phase of normal gait as far as possible, “two step method” was adopted in this study. Although “midgait method” has been widely used in the past, it is limited when applied for diabetes subjects, with the following considerations: (i) diabetic patients have difficulty on accurately reaching the relatively small force plate in a longer step due to impaired coordination caused by neuropathy and possibly associated visual impairment; (ii) many attempts are required to achieve an accurate measurement, which can lead to fatigue that changes the normal gait and testing results; (iii) extensive gait testing also increases the risk of plantar soft tissue damage in diabetic foot patients. McPOIL et al. (47) indicated that through 3~5 times of repeated tests, similar local peak pressure and pressure-time integration could be obtained using the “two-step method” as that measured by “mid-gait method”. Additionally, the DFIS helps the researcher dynamically monitor the thickness change in a 3-dimensional perspective, which could also improve the precision of thickness measuring.

In diabetes patients, a series of chemical reactions would take place between reducing glucose and cellular proteins, which generates advanced glycation end products (AGEs) (48, 49). The accumulation of the AGEs is the major cause of pathophysiologic changes of diabetes tissue (22, 50). Many histomorphometric studies demonstrated thicker, and fragmented fibrous septa, and smaller adipocyte area and diameter in diabetic plantar tissue (10, 16, 17). In this study, we found decreased thickness in diabetes subjects both at time zero (12.25 ± 2.34 mm vs. 14.85 ± 2.81 mm) and following continuous loading (11.95 ± 2.21 mm vs. 14.55 ± 2.74 mm), compared with that of the healthy adults. It could be speculated that this phenomenon is caused by the atrophy of the adipose tissue and degeneration of septa that form the specific honeycomb structure. The peak strain was found to be significantly increased in diabetes patients in our results. This may also be related with the breaking of collagen bundles and fragmentation of elastin strands in septa, which provide elastic constraining force for the fatty chambers. Undoubtedly, the increased deformation during gait makes the heel pad more vulnerable to mechanical trauma and is associated with higher risk of ulceration. Thus, some authors have developed novel methods, such as 3D digital image correlation (3D-DIC) (51, 52), optical coherence tomography (OCT) (53), 3D scanning technology (54), multi-view stereoscopic technology (55) and motion capture system (56), to investigate the surface deformation and strain of plantar tissue. However, these instruments were primarily designed to recorded the surface displacement and strain, and cannot be used for investigating the viscoelastic mechanical characteristics for heel pad.

The cushioning ability of plantar soft tissue is largely depended on the tissue’s viscoelastic characteristic. Thus, if the tissue losses the viscoelasticity due to the continuous accumulation of the AGEs, its capacity of absorbing the shock and uniformly distributing loads during weight bearing activity will be reduced (36). In the previous studies, stiffness has always been the mostly concerned material property of heel pad, and most of them demonstrated increased stiffness for plantar tissue of diabetes patients (9, 28, 35, 40–46). However, in our results, we detected minor decrease of stiffness in diabetes patients compared with that of healthy subjects at time zero, and similar stiffness was found following continuous loading. The slope of loading line in stress-strain curve represents the stiffness, according to modified Kelvin-Voigt viscoelastic model. Thus, the increased magnitude of strain in diabetes patients would be the cause of the decreased stiffness. The significantly negative correlation between stiffness and peak strain in **Figures 4**–**7** could further verify this assumption. What’s more, to eliminate the impact of the age, the subjects in healthy group were matched according to age. As a result, the age in healthy group was relatively older, which consequently would increase the stiffness of this group (57). It is of particular importance to evaluate the time-dependent behaviour (i.e., viscous properties) of heel fat pad, as it has been widely recognized as the major origin of the ability of shock absorption at heel strike (58). What’s more, the modifications on viscous properties may be even more sensitive to pathological conditions, such as diabetes, than other commonly evaluated material properties (such as stiffness) (40). Using our novel system, in consequence, clinicians could easily obtain the viscous parameter to assist the diagnoses and interventions of pathological states at heel. In our results, we demonstrated significantly lower viscous modulus for diabetes patients both at time zero and following continuous loading. Thus, the change on viscous constant can be used as an instructive factor to predict the risk of ulceration., **3**

### Loading history and diabetes course on material properties of heel pad

The history-dependent viscoelastic properties have been widely reported in other soft tissues (59–61). Sommer et al. (59) determined the biaxial extension and triaxial shear properties of the passive human ventricular myocardium, and found that under quasi-static and dynamic multiaxial loadings it is a nonlinear, anisotropic, viscoelastic and history-dependent soft biological material. This study showed clearly higher Cauchy stresses at the same stretch level, caused by history-dependent behaviour (strain softening). The similar strain softening behaviour was also observed during shearing whenever the shear level was increased. Weickenmeier et al. (60) performed a combined experimental and numerical investigation of the mechanical response of superficial facial tissues, and demonstrated location, time and loading history dependent material properties of the facial skin and superficial musculoaponeurotic system. In plantar soft tissue, unfortunately, to date the experiments observing the history-dependent material properties are seldom available. In the present study, we investigated the impact of loading history (continuous weight bearing) on the material characteristics of heel pad, showing decreased primary thickness and viscous modulus both in healthy and diabetes groups. This phenomenon indicates that fatigue status would be related to lowered capacity of shock absorbing with the decreased viscosity, which in turn leads to higher vulnerability to injury and ulceration.

No any significant difference was found when subgroup analysis was perform based on length of diabetes course, however, the regression analysis demonstrated significant positive correlation bewteen the peak strain and length of diabetes history. In study of Sacco et al. (62), they also introduced obvious heterogeneity on the findings about biomechanical characteristics in diabetes, and they speculated that the inconsistent findings may be due to the divergent classification/grouping criteria adopted. Hence, material properties of heel pad in diabetes patients is not uniquely dependent on the length of diabetes course, but many other factors, such as diabetic polyneuropathy, history of ulceration (63) and blooding sugar value (35), should be taken into consideration at the same time. Concerning the impact of dominate leg on the material properties, Flanagan et al. (64) proposed that the dominate leg was related with decreased deformity than non-dominated leg, and speculated that the dominate legs of subjects may contributed to the differences between two legs. While in study of Ugbolue et al. (52), no significant different displacement was evident when comparing the non-dominant and dominant heels. The current study did not found significant differences on material properties between two sides. However, the predominate leg of the participants may be either the left or the right leg, which may cause potential bias on the results. Thus, future researches with large sample size focusing on the influence of dominate leg on heel pad properties is desired.

### Limitations

This study, nevertheless, has some limitations that must be pointed out. Firstly, the strain rate applied to the heel pad has been widely proven to obviously impact the material properties of heel pad (14, 26, 28). While in the stance phase of gait, it is non-possible to precisely control the strain rate as that performed in *in-vitro* machine-based loading. To overcome this problem, subjects were trained prior to measurement to ensure an approximate gait velocity of 1.0 m/s. Then, with the aim of investigating the impacts of diabetes course and side of legs on the material properties, subgroup analyses were conducted. However, the sample sizes (10 legs) in the subgroups are relatively small, which may increase the risk of type II error. Thus, the results of the subgroup analyses should be interpreted with caution at this stage, and some future studies with larger sample size are required to further investigate the impact of diabetes course.

## Conclusions

Utilizing the novel measurement approach, the material properties of heel pad in healthy and diabetes subjects were investigated, in the stance phase of normal gait. As a result, diabetes patients were found to be associated with decreased primary thickness and viscous modulus, and increased peak strain, which may contribute to the vulnerability of heel pad to injury and ulceration. Pre-compression history by continuous weight bearing could significantly lower the primary thickness and viscous modulus of heel pad. Thus, the fatigue status is a risk factor that decrease the cushioning ability of heel pad, which in turn causes ulceration. Among diabetes patients, the length of diabetes course could not be used as the single factor to predict the degree of degeneration, but multiple conditions in diabetes should be taken into consideration.

## Data availability statement

The raw data supporting the conclusions of this article will be made available by the authors, without undue reservation.

## Ethics statement

The studies involving human participants were reviewed and approved by Institutional Review Board of Huashan Hospital, Fudan University. The patients/participants provided their written informed consent to participate in this study.

## Author contributions

XGY, ZLT and XG: methodology, validation, formal analysis, investigation, data curation, writing-original draft, writing-reviewing and editing, and project administration. ZMZ, KW, RH and WMC: investigation, and data processing. CW, LC, CZ, JZH and XW: validation, writing-reviewing and editing. XG, and XM: project administration. All authors contributed to the article and approved the submitted version.

## Funding

This work was supported by grants from the National Natural Science Foundation of China (No. 81902175 & 82172378) and the Shanghai Shen Kang Hospital Development Center (Award number(s): SHDC2020CR3071B). The sponsors or funders had no involvement in any part of this study. All authors confirmed the independence of researchers from funding sources.

## Conflict of interest

The authors declare that the research was conducted in the absence of any commercial or financial relationships that could be construed as a potential conflict of interest.

## Publisher’s note

All claims expressed in this article are solely those of the authors and do not necessarily represent those of their affiliated organizations, or those of the publisher, the editors and the reviewers. Any product that may be evaluated in this article, or claim that may be made by its manufacturer, is not guaranteed or endorsed by the publisher.

## Supplementary material

Supplementary Figure 1 Paired box plot comparing the material properties between left and right foots, before **(A–F)** and after **(G–L)** continuous loading. ##, the difference between two loading statuses follow the normal distribution; $$, the difference between two loading statuses didn’t follow the normal distribution; “t” and “w” represent the statistical effect sizes for paired-T test, and paired-Wilcoxon test, respectively. EDR, energy dissipation rate.

Supplementary Figure 2 The correlation matrix for duration of diabetes history, BMI, age, primary thickness, peak strain, peak stress, stiffness, viscous modulus, and EDR of heel in diabetes subjects at time zero. The values displayed in the right-upper triangle represent the Pearson’s correlation coefficients (R values). The lower-left triangle displays the scatter plots and regression lines. The plots on the diagonal line present the distribution density of the variables in the matrix. BMI: body mass index; EDR, energy dissipation rate. P values:.p<0.100, *p<0.050, **p<0.010, ***p<0.001.

Supplementary Figure 3 The correlation matrix for duration of diabetes history, BMI, age, primary thickness, peak strain, peak stress, stiffness, viscous modulus, and EDR of heel in diabetes subjects following continuous loading. The values displayed in the right-upper triangle represent the Pearson’s correlation coefficients (R values). The lower-left triangle displays the scatter plots and regression lines. The plots on the diagonal line present the distribution density of the variables in the matrix. BMI: body mass index; EDR, energy dissipation rate. P values:.p<0.100, *p<0.050, **p<0.010, ***p<0.001.
