# Supplementary figures and images for: Comparison of material properties of heel pad between adults with and without type 2 diabetes history: An in-vivo investigation during gait
Source: Front Endocrinol (Lausanne). 2022 Aug 17;13:894383. doi: 10.3389/fendo.2022.894383 (PMC9428762; doi:10.3389/fendo.2022.894383)

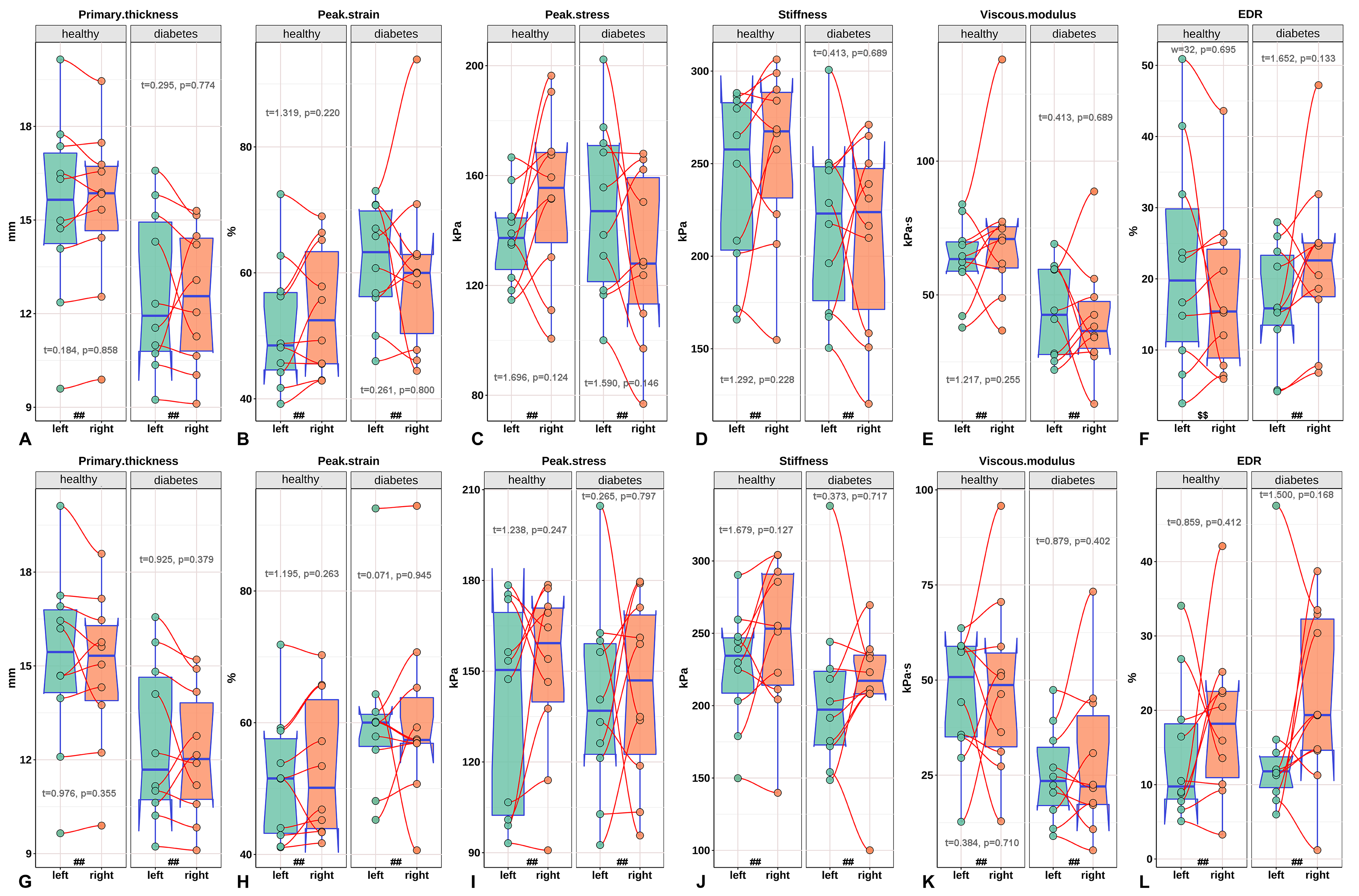

Supplement: Supplementary Figure 1 — Paired box plot comparing the material properties between left and right foots, before (A–F) and after (G–L) continuous loading. ##, the difference between two loading statuses follow the normal distribution; $$, the difference between two loading statuses didn’t follow the normal distribution; “t” and “w” represent the statistical effect sizes for paired-T test, and paired-Wilcoxon test, respectively. EDR, energy dissipation rate. [file Image_1.tif]

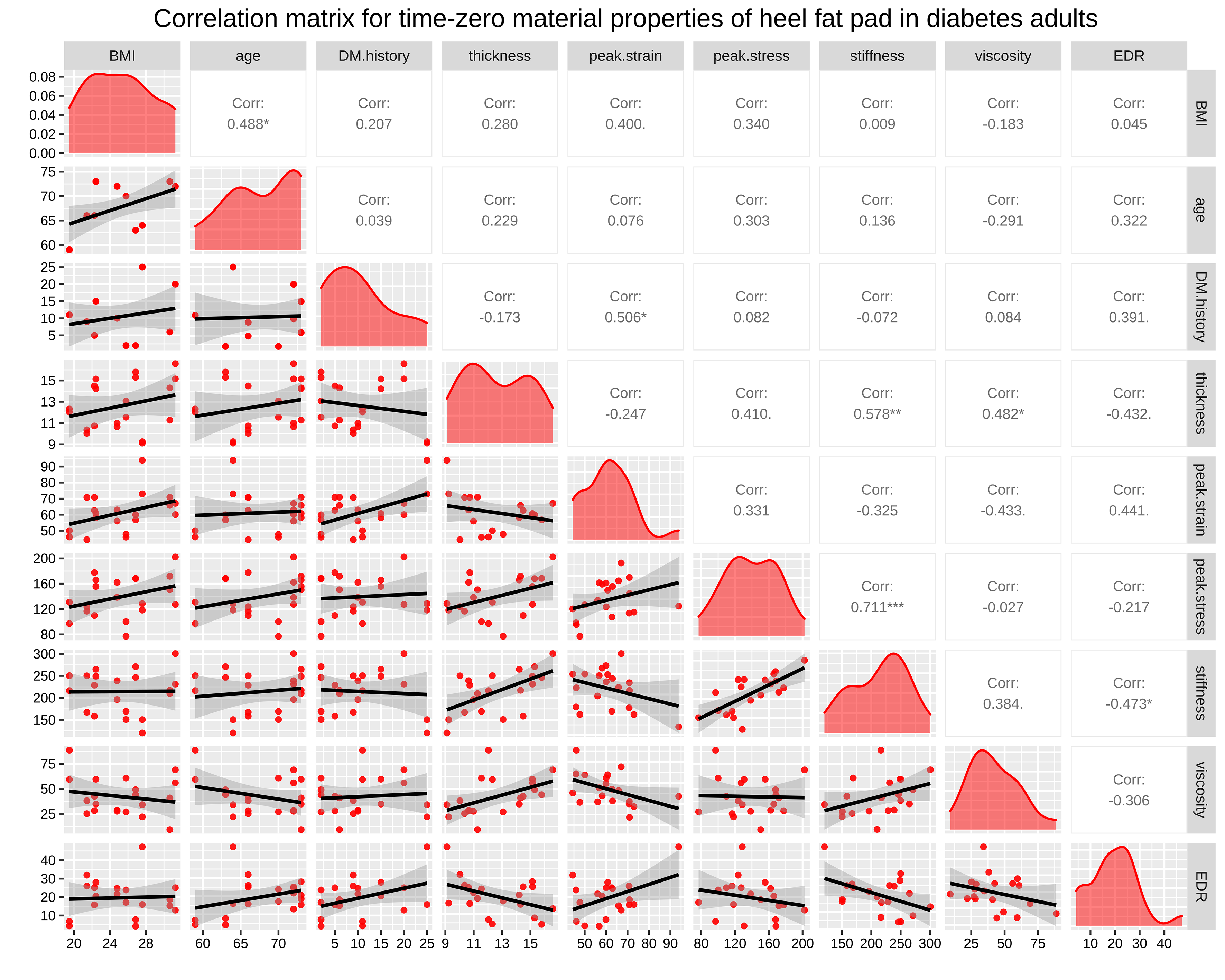

Supplement: Supplementary Figure 2 — The correlation matrix for duration of diabetes history, BMI, age, primary thickness, peak strain, peak stress, stiffness, viscous modulus, and EDR of heel in diabetes subjects at time zero. The values displayed in the right-upper triangle represent the Pearson’s correlation coefficients (R values). The lower-left triangle displays the scatter plots and regression lines. The plots on the diagonal line present the distribution density of the variables in the matrix. BMI: body mass index; EDR, energy dissipation rate. P values:.p<0.100, *p<0.050, **p<0.010, ***p<0.001. [file Image_2.tif]

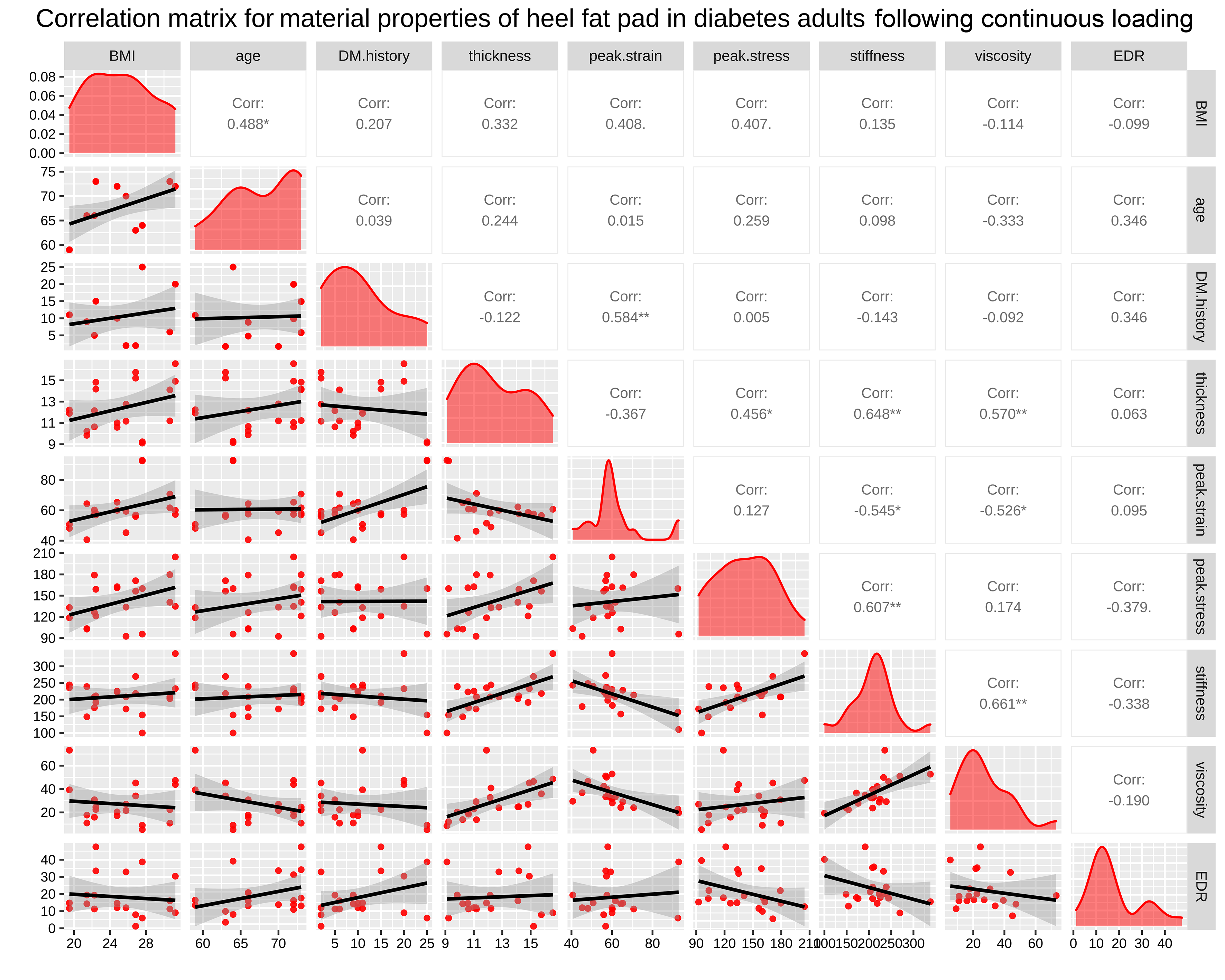

Supplement: Supplementary Figure 3 — The correlation matrix for duration of diabetes history, BMI, age, primary thickness, peak strain, peak stress, stiffness, viscous modulus, and EDR of heel in diabetes subjects following continuous loading. The values displayed in the right-upper triangle represent the Pearson’s correlation coefficients (R values). The lower-left triangle displays the scatter plots and regression lines. The plots on the diagonal line present the distribution density of the variables in the matrix. BMI: body mass index; EDR, energy dissipation rate. P values:.p<0.100, *p<0.050, **p<0.010, ***p<0.001. [file Image_3.tif]
